# Editorial

**Published:** 1892-09

**Authors:** 


### HOMŒOPATHIC PHYSICIAN,

A MONTHLY JOURNAL OF

HOMŒOPATHIC MATERIA MEDICA AND CLINICAL MEDICINE.

"If our school ever gives up the strict inductive method of Hahnemann, we are lost, and deserve only to be mentioned as a caricature in the history of medicine."—CONSTANTINE HERING.

Vol. XII. SEPTEMBER, 1892. No. 9.

### EDITORIAL.

THE PROCEEDINGS OF THE I. H. A.—Last month was published in this journal the first instalment of the minutes of the meeting of the International Hahnemannian Association at Narragansett Pier, in June. It consisted of the President's address. This masterly production will commend itself to all our readers for its clear setting forth of the position of Homœopathy to-day.

*We are right and we know it*, is the confident and fearless assertion of the President.

All of us who have had difficult and discouraging cases; and have spent many a weary hour in the search for the simillimum; and having found it, have administered it; and then with the patient's agony and possible death confronting us, have calmly waited for the response of reviving nature and been rewarded by a striking cure; all of us with the joy of this experience before us will heartily re-echo the words of the President, "*we are right and we know it.*"

Another section of the proceedings is gived in this number, and we shall continue thus until the whole session has been laid before our readers.

The proceedings were stenographically reported by Dr. J. B.

S. King, of Chicago, associate editor of *The Medical Advance*, and it is his report which we shall use.

---

THE HERING COLLEGE.—Still another new college has been added to the number of medical schools in Chicago. It claims to teach only pure Homœopathy. Unfortunately the new college has a very serious obstacle to overcome.

It has aroused the enmity of the Illinois State Board of Health, and that institution seems as if it wished to destroy it if possible. For an account of the causes of this enmity, the reader is referred to the editorial to be published in September number of *The Medical Visitor*, and which we have been permitted to copy in our pages.

